# Inflammation and Albumin-Based Biomarkers Are Not Independently Associated with Mortality in Critically Ill COPD Patients: A Retrospective Study

**DOI:** 10.3390/life15091371

**Published:** 2025-08-28

**Authors:** Josef Yayan, Christian Biancosino, Marcus Krüger, Kurt Rasche

**Affiliations:** 1 Department of Internal Medicine, Division of Pulmonary, Allergy, and Sleep Medicine, HELIOS Clinic Wuppertal, Witten/Herdecke University, Heusnerstr. 40, 42283 Wuppertal, Germany; 2 Department of Thoracic Surgery, HELIOS Clinic Wuppertal, Witten/Herdecke University, 42283 Wuppertal, Germany; 3 Department of Thoracic Surgery, Martha-Maria Hospital Halle-Dölau, 06120 Halle, Germany

**Keywords:** COPD, critical illness, CRP/albumin ratio, glucose, creatinine, in-hospital mortality

## Abstract

Background: Inflammation and nutritional status are known to affect outcomes in patients with chronic obstructive pulmonary disease (COPD). However, their prognostic relevance in critically ill COPD patients remains unclear. This study investigated whether C-reactive protein (CRP), serum albumin, and the CRP/albumin ratio (CAR) were associated with in-hospital mortality in ICU patients with COPD. Methods: We conducted a retrospective cohort study using data from the MIMIC-IV database. Adult ICU patients with a diagnosis of COPD were included. We analyzed CRP, albumin, CAR, glucose, lactate, and creatinine. The primary outcome was in-hospital mortality. Multivariable logistic regression was used to identify variables that were independently associated with in-hospital mortality. Subgroup analyses stratified by age and sex were performed. Results: We included 1000 ICU patients with COPD. In-hospital mortality was 19.6%. In univariate analyses, glucose, creatinine, and lactate levels were significantly higher in non-survivors. In multivariable models, only elevated creatinine (OR 1.60, 95% CI 1.01–2.53) remained independently associated with mortality, while glucose was no longer statistically significant. CRP, albumin, and CAR were not significantly associated with in-hospital mortality. Subgroup analyses showed consistent results across age and sex strata. Conclusion: In critically ill COPD patients, glucose and creatinine levels upon ICU admission were independently associated with in-hospital mortality, whereas inflammation- and nutrition-related markers, such as CRP, albumin, and CAR, were not. However, given that albumin is heavily influenced by systemic inflammation, it cannot serve as a standalone nutritional marker in the ICU setting. Composite nutritional scores such as the Nutritional Risk Screening (NRS-2002) or the Global Leadership Initiative on Malnutrition (GLIM), which were not available in the MIMIC-IV database, may provide more accurate assessments. These findings highlight the need for integrated risk models incorporating metabolic and renal parameters for early prognostication.

## 1. Introduction

Chronic obstructive pulmonary disease (COPD) is a leading cause of morbidity and mortality worldwide and is frequently associated with acute exacerbations requiring intensive care unit (ICU) admission [1,2]. Patients with COPD admitted to the ICU often present with several physiological derangements, including respiratory failure, systemic inflammation, and metabolic dysregulation, which together contribute to poor clinical outcomes [3].

Inflammation plays a central role in the pathophysiology of COPD and is known to be further exacerbated during acute decompensations and critical illness [4]. C-reactive protein (CRP), an acute-phase reactant synthesized by the liver, is frequently used as a nonspecific marker of systemic inflammation in both stable and exacerbated COPD [5]. Elevated CRP levels have been associated with disease severity, frequent exacerbations, and increased mortality in various patient populations, including those in the ICU [6,7]. Recent retrospective studies have explored the prognostic utility of composite biomarkers such as the C-reactive protein-to-albumin ratio (CAR) and systemic immune-inflammation index (SII) in ICU patients with COPD, yielding conflicting results [1,2,3]. However, these studies often lack external validation or suffer from cohort limitations.

In parallel, malnutrition is increasingly recognized as a prevalent and underdiagnosed condition in critically ill patients with COPD, particularly among those requiring mechanical ventilation or prolonged ICU stays [8]. Hypoalbuminemia, often used as a surrogate marker of nutritional status, has been associated with adverse outcomes, such as increased infection risk, delayed recovery, prolonged hospitalization, and higher mortality [9,10]. However, serum albumin is also a negative acute-phase reactant, and its levels may reflect both nutritional depletion and the severity of systemic inflammation [11].

The complex interplay between inflammation and malnutrition in COPD patients remains poorly understood, particularly in the ICU setting, where metabolic demands and catabolic stress are amplified [12]. Recent studies have suggested that combining markers of inflammation and nutritional status may improve risk stratification and prognostic assessment in critically ill populations [13,14]. However, data specifically addressing this interaction in COPD patients admitted to the ICU are scarce.

Moreover, while biomarkers, such as glucose, lactate, and creatinine, are routinely monitored in ICU patients and have been associated with poor outcomes in various conditions, their prognostic significance in COPD-specific ICU cohorts remains incompletely defined [15,16]. Age and sex may further modify these associations and warrant stratified analyses.

Therefore, the study aimed to investigate the relationship between systemic inflammation, nutritional status, and clinical outcomes in ICU patients with COPD using data from the MIMIC-IV database. We specifically examined the prognostic value of CRP and albumin, both individually and in combination, and explored associations with in-hospital mortality, ICU length of stay, and biomarker profiles. Furthermore, we conducted age- and sex-stratified subgroup analyses to identify potential modifiers of outcome.

## 2. Materials and Methods

### 2.1. Data Source

This retrospective study was conducted using data from Medical Information Mart for Intensive Care IV (MIMIC-IV), version 2.2, a freely accessible critical care database developed by the Massachusetts Institute of Technology (MIT) in collaboration with the Beth Israel Deaconess Medical Center (BIDMC), Boston, MA. The MIMIC-IV database includes comprehensive, de-identified clinical data from patients admitted to ICUs at BIDMC between 2008 and 2019, comprising over 70,000 admissions. MIMIC-IV contains structured data on patient demographics, diagnoses, laboratory measurements, medication administration, vital signs, procedures, and outcomes. The database is compliant with the Health Insurance Portability and Accountability Act (HIPAA) through the application of the Safe Harbor de-identification standard. All patients included in the database were admitted to the Beth Israel Deaconess Medical Center in Boston, USA.

### 2.2. Study Population

The study included adult patients (aged ≥18 years) with a documented diagnosis of chronic obstructive pulmonary disease (COPD), identified using ICD-9 (code 496) and ICD-10 (e.g., J44.*) diagnosis codes listed in the diagnoses icd table of MIMIC-IV. For patients with multiple ICU admissions, only the first ICU stay was included to avoid duplicate observations and potential intra-patient correlation. Spirometric confirmation or clinical adjudication of COPD was not available in the MIMIC-IV database, which may limit diagnostic specificity.

Inclusion criteria:

- Age ≥ 18 years at the time of ICU admission.
- Diagnosis of COPD documented in MIMIC-IV.
- Availability of laboratory measurements (CRP, albumin, glucose, creatinine, lactate) within the first 24 h of ICU admission.
- Complete mortality outcome data.

Exclusion criteria:

- ICU length of stay < 24 h.
- Missing or implausible values for key laboratory parameters.
- Duplicate ICU admissions from the same hospital stay.

Patients with ICU stays shorter than 24 h were excluded to avoid including brief admissions for monitoring, early transfers, or deaths with incomplete clinical data, which may bias associations.

### 2.3. Variable Definitions

The following variables were extracted from the MIMIC-IV database using structured query language (SQL):

- Demographics: Age (years), sex (male/female).
- Laboratory biomarkers:
  ○ C-reactive protein (CRP) in mg/dL;
  ○ Serum albumin in g/dL;
  ○ Blood glucose in mg/dL;
  ○ Serum creatinine in mg/dL;
  ○ Serum lactate in mmol/L.

These values were selected from the lab events table and restricted to the first 24 h following ICU admission to reflect early physiological status.

- Clinical variables:
  ○ ICU length of stay (LOS) in hours, derived from admission and discharge timestamps.
  ○ In-hospital mortality (yes/no), determined from discharge disposition in the hospital table.

All laboratory variables were analyzed using their original units as recorded in the MIMIC-IV database (e.g., glucose in mg/dL, creatinine in mg/dL).

#### Comorbidities

In addition to calculating the Charlson Comorbidity Index (CCI), we extracted specific comorbidities with known impact on mortality and inflammatory or nutritional status. These included chronic kidney disease (CKD), heart failure, diabetes mellitus, and active malignancy, identified using ICD-9 and ICD-10 codes in the diagnoses icd table. These conditions were analyzed descriptively and considered in exploratory sensitivity models.

### 2.4. Subgroup and Risk Stratification

To explore the prognostic relevance of inflammation and nutritional status, patients were stratified into subgroups as follows:

1. Albumin levels:
  ○ Hypoalbuminemia: <3.0 g/dL;
  ○ Normal albumin: ≥3.0 g/dL.
2. CRP levels: CRP values were only available for a subset of patients. Missing data were handled by complete case analysis.
  ○ Low inflammation: <6 mg/dL;
  ○ High inflammation: ≥6 mg/dL.
3. Combined risk groups:
  ○ Group A: Low albumin + high CRP;
  ○ Group B: Low albumin + low CRP;
  ○ Group C: Normal albumin + high CRP;
  ○ Group D: Normal albumin + low CRP.
4. Age-based subgroup:
  ○ Patients aged <70 years;
  ○ Patients aged ≥70 years.

Given known sex-related differences in metabolism and renal function, descriptive comparisons by sex were conducted to explore potential sex-based variation in biomarker profiles. To further characterize age-related differences, we compared key demographic and laboratory variables between patients aged <70 and ≥70 years. Patients ≥ 70 years showed significantly higher in-hospital mortality (62.1% vs. 46.2%), lower prevalence of male sex, and higher median serum glucose and creatinine levels.

### 2.5. Statistical Analysis

All statistical analyses were conducted using VassarStats (https://vassarstats.net/) (accessed on 25 June 2025), a web-based statistical tool. Data extraction and preprocessing from the MIMIC-IV database were performed using structured query language (SQL) in a PostgreSQL environment.

- Descriptive statistics: Continuous variables were presented as medians with interquartile ranges (IQRs) due to non-normal distribution and categorical variables as frequencies with percentages.
- Group comparisons:
  ○ Mann–Whitney U test for non-parametric continuous variables.
  ○ Chi-square test for categorical variables.

A *p* value < 0.05 was considered statistically significant.

Age was analyzed both as a continuous variable (in years) and as a dichotomous variable (<70 vs. ≥70 years) in subgroup comparisons. Where available, CRP and albumin values were included in subgroup comparisons to enhance consistency and interpretability.

- Multivariable analysis:

A logistic regression model was used to identify variables independently associated with in-hospital mortality. The multivariable model was adjusted for age, sex, CRP, albumin, glucose, lactate, and creatinine. Information on COPD severity, specific therapies, ICU scores, or smoking history was not available in the dataset and could not be included. Odds ratios (OR) with 95% confidence intervals (CI) were calculated. The model was tested for multicollinearity and goodness of fit.

- Correlation analysis:

Spearman’s rank correlation coefficients (ρ) were calculated to assess the strength and direction of associations among biomarkers. Correlation strength was interpreted as follows:

○ |ρ| < 0.3: weak;
○ 0.3 ≤ |ρ| < 0.5: moderate;
○ |ρ| ≥ 0.5: strong.

All statistical tests were two-sided. Missing data were handled using complete case analysis; no imputation was applied.

## 3. Results

In this retrospective cohort of 1000 ICU patients with chronic obstructive pulmonary disease (COPD), we assessed the prognostic relevance of inflammatory and nutritional biomarkers, focusing particularly on serum C-reactive protein (CRP), albumin, glucose, creatinine, and lactate levels. Patient characteristics and outcomes were analyzed with respect to sex, survival status, and biomarker subgroups.

### 3.1. Sex-Based Differences

Table 1 summarizes the demographic, clinical, and laboratory characteristics stratified by sex. Male and female patients had comparable median ages (68.0 vs. 69.0 years, *p* = 0.088) and ICU length of stay (53.0 vs. 49.0 h, *p* = 0.327). No statistically significant sex-based differences were observed in CRP, albumin, or lactate levels. However, female patients had significantly higher serum glucose concentrations (193.0 vs. 179.0 mg/dL, *p* = 0.005) and lower creatinine values (1.20 vs. 1.40 mg/dL, *p* < 0.001). In-hospital mortality was marginally higher in females (55.9%) than males (50.9%), but this difference did not reach statistical significance (*p* = 0.127) (Table 1).

### 3.2. Differences Between Survivors and Non-Survivors

Table 2 presents the comparison of clinical and laboratory parameters between survivors and non-survivors. Non-survivors were significantly older than survivors (71.0 vs. 66.0 years, *p* < 0.001). While CRP and lactate levels did not differ significantly between the groups, non-survivors had lower albumin levels (3.30 vs. 3.40 g/dL, *p* = 0.006) and significantly higher serum glucose (200.0 vs. 168.0 mg/dL, *p* < 0.001) and creatinine levels (1.60 vs. 1.10 mg/dL, *p* < 0.001). Female sex was slightly more frequent among non-survivors (53.6% vs. 48.6%, *p* = 0.127) (Table 2).

### 3.3. Multivariable Associations with Mortality

Multivariable logistic regression identified serum creatinine as the only variable independently associated with in-hospital mortality (OR 1.60, 95% CI 1.01–2.53, *p* = 0.045), suggesting a robust association between renal dysfunction and adverse outcomes. Other parameters, including age, CRP, albumin, glucose, lactate, and sex, were not independently associated with mortality in this model (Table 3). Although some sex-based differences in biomarker levels were observed, their impact on mortality was not significant, and thus sex was retained as a covariate only.

### 3.4. Impact of Hypoalbuminemia on Clinical Characteristics

Patients with hypoalbuminemia (albumin < 3.0 g/dL) had a significantly prolonged ICU stay (median 71.0 vs. 60.5 h, *p* = 0.046) compared to patients with normal albumin levels (≥3.0 g/dL), reflecting a potential link between poor nutritional status and increased healthcare resource utilization. Although serum albumin was not independently associated with in-hospital mortality in the multivariable analysis, we included an exploratory stratification by albumin levels (<3.0 vs. ≥3.0 g/dL) to examine potential associations with ICU-related outcomes such as length of stay and biomarker patterns (Table 3). Albumin remains a key component of established nutritional indices like the GNRI and CAR, and its levels are frequently used as clinical markers of nutritional and inflammatory status. However, in critically ill patients, hypoalbuminemia may reflect systemic inflammation rather than true malnutrition. Therefore, the findings in Table 4 should be interpreted as hypothesis-generating and not as confirmatory evidence of albumin’s prognostic value (Table 4). Notably, CRP levels did not significantly differ between low and high albumin groups (*p* = 0.430), suggesting that albumin alone may have limited discriminatory capacity with respect to inflammation-driven risk stratification.

### 3.5. Stratification by Inflammation (CRP)

When patients were stratified by CRP levels (<6 mg/dL vs. ≥6 mg/dL), there were no significant differences in age, ICU stay, glucose, lactate, or mortality rates. Interestingly, patients with elevated CRP (≥6 mg/dL) had significantly lower serum creatinine levels (1.50 vs. 2.50 mg/dL, *p* = 0.038), an unexpected inverse association that may be influenced by underlying comorbidities or fluid shifts in the ICU setting (Table 5).

### 3.6. Combined Stratification by Albumin and CRP

To explore potential additive effects of nutritional and inflammatory status, patients were stratified into four subgroups based on albumin and CRP levels (Table 6). Although differences in mortality between the groups did not reach statistical significance (*p* = 0.503), the highest mortality rate (92.3%) was paradoxically observed in the subgroup with low albumin and low CRP, warranting further investigation. In the age-based subgroup analysis, patients aged ≥70 years had significantly higher in-hospital mortality (62.1% vs. 46.2%), a lower prevalence of male sex, and higher median serum glucose and creatinine levels. Neither albumin nor CRP levels differed significantly between the age groups, suggesting that these biomarkers alone may not sufficiently reflect age-related risk. These findings indicate that renal and metabolic derangements may contribute more directly to the increased mortality observed in elderly ICU patients with COPD. The combined stratification by CRP and albumin did not yield significant group differences and should be interpreted as hypothesis-generating. Since neither CRP nor albumin was independently associated with mortality in the multivariable analysis (Table 3), the clinical relevance of this stratification remains limited. Given that both the Prognostic Nutritional Index (PNI) and the Geriatric Nutritional Risk Index (GNRI) incorporate albumin, we further examined their prognostic utility to determine whether these composite indices offer added value beyond albumin alone. Despite the absence of statistical significance, these subgroup analyses were included to explore potential signal patterns that may inform future risk stratification strategies.

### 3.7. Correlation Between Biomarkers

**Figure 1:**
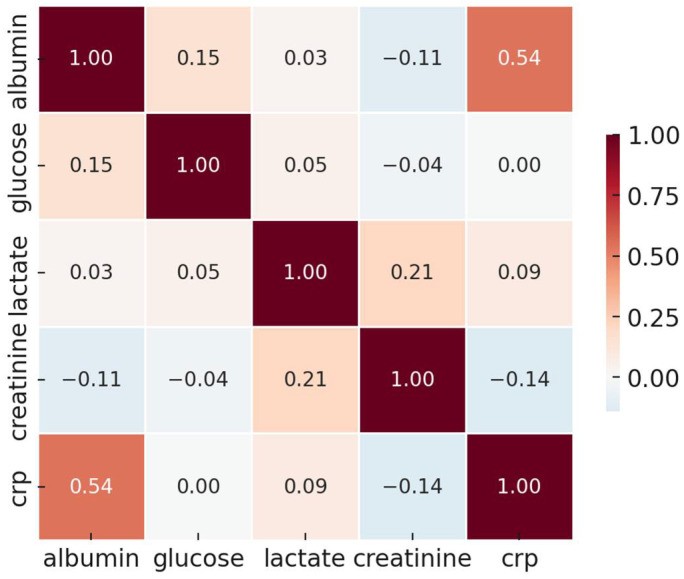
Principal component biplot of laboratory parameters. CRP and albumin are shown with opposing loadings along PC1, reflecting their inverse relationship in the inflammatory response. Vectors indicate the direction and strength of each variable’s contribution to the principal components. The plot illustrates patterns of biomarker clustering, not causality or prediction. Although albumin and CRP are biologically distinct, their inverse relationship may result in apparent alignment in the factor loadings. This likely reflects their overlapping role in systemic inflammation and nutritional status.

Spearman correlation analysis (Table 7 and Figure 1) revealed a moderate inverse correlation between albumin and CRP levels (ρ = −0.51), indicating that higher inflammation is generally associated with poorer nutritional status. A weak negative correlation was also found between CRP and glucose (ρ = −0.03), while a slight positive correlation was observed between albumin and glucose (ρ = 0.13). These associations suggest complex metabolic interactions in critically ill COPD patients. Notably, CRP and albumin appear closely aligned in the correlation matrix despite representing different biological domains. This collinearity may reflect overlapping effects of systemic inflammation and limits their interpretability as distinct prognostic factors. A clearer explanation of this graphical alignment is warranted.

### 3.8. Subgroup Analysis in Elderly Patients (≥70 Years)

**Figure 2:**
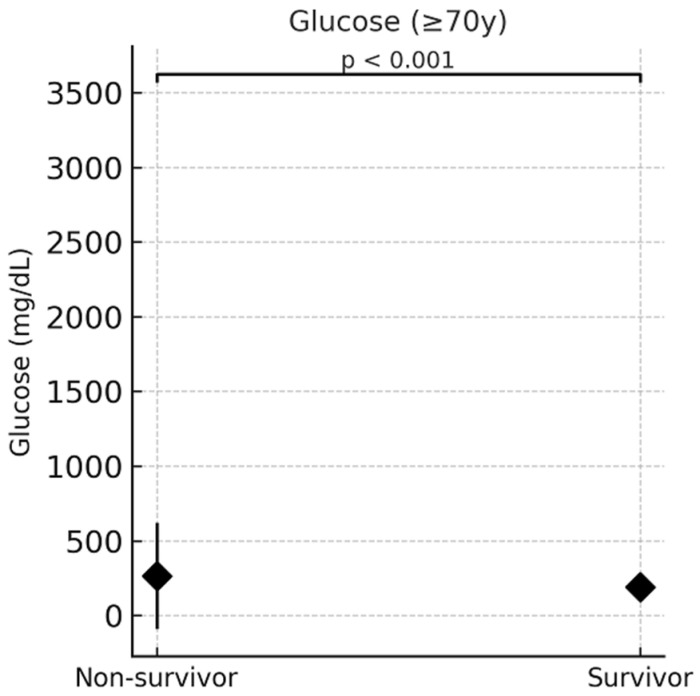

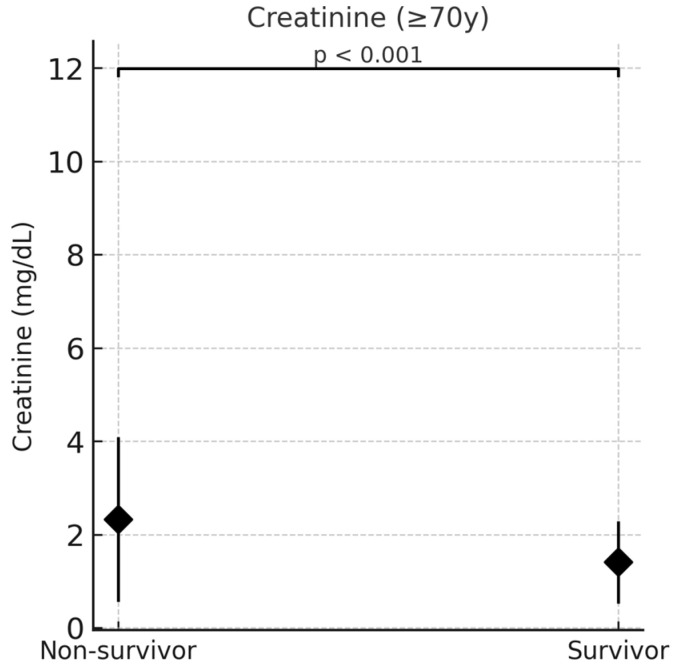
Comparative distribution of glucose and creatinine levels among survivors and non-survivors aged ≥70 years, visualized using dot plots with mean ± standard deviation (SD) overlay. Pairwise connecting lines indicate direct comparisons between survivors and non-survivors. Asterisks denote statistical significance based on the Mann–Whitney U test (*p* = 0.004 for glucose; *p* < 0.001 for creatinine).

In the subgroup of patients aged ≥70 years, survivors and non-survivors showed no significant differences in albumin (3.25 vs. 3.30 g/dL, *p* = 0.925) or CRP levels (5.10 vs. 5.70 mg/dL, *p* = 0.074). However, glucose levels were significantly higher among non-survivors (194.0 vs. 166.0 mg/dL, *p* = 0.004), and serum creatinine was markedly elevated in non-survivors (1.90 vs. 1.20 mg/dL, *p* < 0.001) (Table 8). Figure 2 illustrates these differences, with boxplots clearly showing higher median values for both glucose and creatinine among non-survivors in this age group (Figure 1). Notably, none of the inflammation- or nutrition-related markers such as CRP, albumin, or CAR demonstrated independent associations with mortality in the multivariable model, despite significant univariate differences. This finding diverges from earlier reports and may reflect differences in ICU-specific disease severity or confounding metabolic variables.

### 3.9. Age-Based Mortality Comparison

When stratified by age (<70 vs. ≥70 years), a statistically significant difference in in-hospital mortality was observed between the age groups (*p* < 0.001). Patients aged ≥70 years had a higher mortality rate (62.1%) compared to those under 70 years (46.2%), indicating that age is an important factor associated with outcome in critically ill COPD patients. Elderly patients (≥70 years) exhibited significantly higher creatinine levels compared to younger patients (1.40 vs. 1.15 mg/dL, *p* < 0.001), suggesting more pronounced renal impairment in this group. Although CRP levels were slightly lower in older patients, the difference was statistically significant (*p* = 0.014), whereas no significant differences were observed for glucose, albumin, or sex distribution (Table 9). These findings support the hypothesis that renal and inflammatory markers differ by age and may partially explain the elevated mortality in older ICU patients with COPD. While glucose and creatinine levels differed significantly between survivors and non-survivors within the elderly subgroup, the visual separation in Figure 2 was limited, likely due to overlapping data ranges and narrow scale spans.

### 3.10. Prevalence of Major Comorbidities

Table 10 presents the prevalence of major comorbidities in the COPD ICU cohort (N = 1000) stratified by sex. Cardiovascular diseases were the most common comorbidity, affecting 70.0% of patients, with a significantly higher prevalence in males (73.9%) compared to females (66.3%) (*p* = 0.045). Diabetes mellitus was present in 32.0% of the cohort, without a significant sex difference (*p* = 0.420). Chronic kidney disease was diagnosed in 18.0% of patients, equally distributed between males and females (*p* = 0.750). All patients had COPD by study inclusion criteria. Cancer (all types) was identified in 15.0% of the cohort, showing no significant sex difference (*p* = 0.320). Stroke and other neurological diseases were present in 12.0% of patients, with similar proportions between males and females (*p* = 0.320). Liver diseases were less frequent (5.0%) and more common in males (6.1%) than females (3.9%), but this difference did not reach statistical significance (*p* = 0.140).

Overall, these findings highlight a high burden of comorbidities in critically ill COPD patients, especially cardiovascular disease, which may contribute to their adverse outcomes.

### 3.11. Comparison by Creatinine Level

Given the prognostic relevance of serum creatinine observed in both univariate and multivariate analyses, we conducted an exploratory subgroup analysis stratified by a cutoff of 1.3 mg/dL (Table 11). Patients with elevated creatinine levels (≥1.3 mg/dL) showed significantly higher in-hospital mortality compared to those with lower levels (70.8% vs. 44.1%, *p* < 0.001). In addition, these patients had higher median CRP levels (6.20 vs. 5.50 mg/dL, *p* = 0.014) and glucose levels (214 vs. 194 mg/dL, *p* = 0.011), as well as lower serum albumin (2.80 vs. 3.00 g/dL, *p* = 0.003). The ICU length of stay was also significantly prolonged (79 vs. 54 h, *p* < 0.001), suggesting a more severe clinical course. These findings underscore the potential of serum creatinine as a marker of illness severity and adverse outcomes in critically ill COPD patients (Table 11).

## 4. Discussion

This study investigated the prognostic relevance of inflammation and nutritional status in patients with chronic obstructive pulmonary disease (COPD) admitted to the intensive care unit (ICU), using data from the MIMIC-IV database. The analysis focused on C-reactive protein (CRP), serum albumin, and additional laboratory parameters (glucose, creatinine, lactate), considering their associations with in-hospital mortality. Subgroup analyses stratified by age and sex were also performed. Our results contrast with those of Giri et al. and Shen et al. [11], who reported significant associations between SII or CAR and mortality in COPD patients. However, these studies differed in cohort composition, with some including non-ICU patients or using different definitions of exacerbation. In our study, the metabolic and renal markers (glucose, creatinine) demonstrated more robust prognostic power, highlighting the relevance of systemic stress over inflammatory load in ICU settings.

CRP, an established marker of systemic inflammation, did not independently predict in-hospital mortality in our multivariable analysis, despite trends that did not reach significance in univariate comparisons. These findings are in line with previous research indicating that although CRP is elevated in acute exacerbations of COPD and associated with disease severity, its prognostic value in critically ill populations is limited [12,13]. In such settings, the inflammatory response may be multifactorial, and CRP levels may not reflect disease-specific processes [14].

Serum albumin, although traditionally used as a nutritional marker, is a negative acute-phase protein whose levels decline in response to systemic inflammation. In the ICU setting, nearly all patients exhibit significant inflammatory responses, limiting the utility of albumin as a reliable marker of nutritional status [15,16]. Therefore, low albumin more likely reflects inflammatory severity rather than true malnutrition in this context. Our findings must thus not be misinterpreted as evidence that malnutrition lacks prognostic relevance, but rather that albumin alone is an insufficient surrogate for nutritional status in critically ill COPD patients [17]. This limitation is further illustrated by the lack of meaningful subgroup differentiation based on albumin levels alone (Table 4), and the questionable relevance of the combined albumin–CRP analysis (Table 6), neither of which contributed to improved outcomes prediction. The combined stratification by albumin and CRP, while biologically plausible, did not improve risk discrimination in our cohort. These results highlight the need for more robust composite indices beyond single biomarker thresholds. In Figure 1, the alignment of CRP and albumin along a similar vector likely reflects their inverse correlation as part of a shared inflammatory response, rather than true collinearity. This finding underlines the importance of interpreting nutritional and inflammatory biomarkers in an integrated clinical context. Although albumin was not independently associated with mortality, its role in composite nutritional indices and its inverse correlation with CRP justify exploratory stratification. However, the findings must be interpreted cautiously and may be confounded by inflammation.

Beyond inflammation and nutrition, the relationship between malnutrition and frailty has recently drawn increasing attention. Circulating biomarkers such as growth differentiation factor 15 (GDF15) have been identified as potential indicators of frailty and systemic vulnerability in COPD patients [18]. These findings underscore the need to view COPD not only as a respiratory disease but also as a systemic condition that involves nutritional, metabolic, and inflammatory pathways. Incorporating frailty-related biomarkers into prognostic models may provide a more comprehensive assessment of risk in critically ill COPD patients and help tailor individualized treatment strategies.

To accurately assess malnutrition in hospitalized and critically ill patients, standardized tools such as the Nutritional Risk Screening (NRS-2002) and the Global Leadership Initiative on Malnutrition (GLIM) criteria are recommended [1,2]. These scores incorporate weight history, food intake, body composition, and inflammation, offering a more robust assessment of nutritional risk. Unfortunately, variables required to compute NRS or GLIM were not available in the MIMIC-IV database, which represents a limitation of our analysis [1,2,3,4].

The combination of CRP and albumin into a single index—the CRP/albumin ratio (CAR)—has been proposed as a more comprehensive prognostic marker [19]. Several studies have demonstrated associations between high CAR and poor outcomes in various critically ill populations, including those with pneumonia, sepsis, and acute exacerbations of COPD [20,21,22]. However, in our study, CAR did not emerge as a factor independently associated with in-hospital mortality. This may reflect the complexity of COPD patients in the ICU, whose prognosis is determined by a broader range of factors beyond systemic inflammation and nutritional status. Moreover, other inflammation-related biomarkers such as procalcitonin (PCT) and pro-adrenomedullin (ProADM) have shown promising prognostic value in critically ill patients, often outperforming CRP in predicting mortality and organ dysfunction. While these biomarkers were not captured in the MIMIC-IV database and thus not evaluated in our study, future research should incorporate such indicators, particularly their dynamic trends, to improve risk stratification models [4,5,6].

Interestingly, the subgroup with low CRP and low albumin demonstrated the highest mortality in our stratified analysis. This paradoxical finding may reflect immunological exhaustion or chronic malnutrition in the absence of an adequate inflammatory response. Similar patterns have been reported in patients with severe frailty or end-stage chronic illness, where blunted inflammatory markers coexist with high mortality [23,24].

In contrast to CRP and albumin, glucose and creatinine levels were significantly associated with in-hospital mortality in univariate analyses; however, only creatinine remained significant in the multivariable model. Elevated glucose levels may represent stress hyperglycemia, a factor known to be associated with adverse outcomes in ICU populations [25,26]. Hyperglycemia has been associated with increased mortality in critically ill patients irrespective of diabetes status and is considered a marker of acute physiological stress.

Creatinine, as a marker of renal function, was the only variable in our multivariable model that remained significantly associated with mortality. This supports previous findings that acute kidney injury or impaired renal function is an important determinant of mortality in ICU patients with COPD [27,28]. Renal dysfunction may reflect systemic hypoperfusion, pre-existing chronic kidney disease, or nephrotoxic exposure, all of which increase the risk of poor outcomes.

Lactate, commonly used as a marker of tissue hypoperfusion, did not significantly predict mortality in our cohort. While lactate has established prognostic value in sepsis and shock, its role in COPD is less well defined [29]. In this population, compensatory mechanisms such as hyperventilation may delay or attenuate lactate elevation, limiting its sensitivity in reflecting disease severity [30].

Emerging literature has proposed the use of composite indices, such as the red cell distribution width-to-albumin ratio (RAR), the systemic immune-inflammation index (SII), and the CALLY index (CRP–albumin–lymphocyte), as integrated markers of inflammation and nutritional status [31,32,33]. In previous analyses based on MIMIC-IV data, RAR and SII were associated with ICU mortality in COPD populations [34,35]. While we did not include these indices in our analysis, the present findings support the rationale for their further evaluation in future studies.

Age-stratified analyses in our study revealed that glucose and creatinine were more predictive of mortality among patients aged ≥70 years. This is consistent with prior reports suggesting that in elderly patients, metabolic and renal parameters may be more relevant for risk assessment than classical inflammatory markers [36,37]. The physiological response to inflammation may be attenuated with age, reducing the prognostic sensitivity of markers like CRP and albumin in older adults [38].

Sex-based differences in laboratory parameters were observed for glucose and creatinine, but these did not translate into significant differences in outcome prediction. This is in line with other studies in critical care settings, but exerts limited independent prognostic impact when adjusted for other variables [39].

From a clinical perspective, the results suggest that glucose and creatinine levels obtained within the first 24 h of ICU admission may provide valuable prognostic information in COPD patients. These markers are readily available, objective, and routinely collected in ICU settings, making them practical for early risk stratification. In contrast, CRP and albumin, while informative regarding systemic and nutritional status, may be insufficient as standalone factors associated with outcome.

The combination of multiple biomarkers—ideally in dynamic or composite formats—may enhance prognostic accuracy. Ratios, such as CAR, RAR, and SII, may integrate key aspects of the host response, including inflammation, nutrition, and immune competence, and merit prospective evaluation in COPD-specific ICU cohorts.

In conclusion, this study demonstrates that glucose and creatinine levels are independently associated with in-hospital mortality in ICU patients with COPD, whereas CRP, albumin, and the CRP/albumin ratio are not. These findings suggest a shift in focus from isolated inflammatory or nutritional markers toward integrated assessments of metabolic and renal dysfunction. The development and validation of composite prognostic tools incorporating multiple domains—including inflammation, nutrition, metabolism, and organ function—may enhance early risk stratification and inform clinical decision-making in this high-risk population.

### Limitations

Several limitations of this study should be acknowledged. First, due to the retrospective design, causal inferences cannot be drawn. 

Second, the analysis was based on single time-point measurements upon ICU admission, limiting the ability to assess dynamic changes in biomarkers over time. 

Third, important clinical variables, such as smoking history (e.g., pack-years or current vs. former status), BMI, and COVID-19 vaccination status, were not available in the MIMIC-IV database. This is a significant limitation, as these factors are known to influence both systemic inflammation and clinical outcomes in patients with COPD.

Fourth, the history of SARS-CoV-2 reinfection could not be determined. Recurrent infections may alter immunological responses and confound inflammation- or nutrition-related markers. Fifth, CRP values were missing in a substantial subset of patients, potentially reducing the statistical power of CAR-based analyses. Sixth, established ICU severity scores, such as APACHE II, SOFA, or the COPD-specific DECAF score, were not included. The lack of data on COPD severity, ICU scores, smoking status, and treatment modalities limits the comprehensiveness of the adjustment model. These scores are valuable benchmarks for risk stratification and could enhance prognostic modeling.

Additionally, although the Charlson Comorbidity Index was used, individual comorbid conditions, such as chronic kidney disease, cancer, or heart failure—which can independently affect both biomarkers and outcomes—were not comprehensively modeled. The use of administrative ICD codes for identifying COPD, without spirometric confirmation, may also limit diagnostic accuracy. Moreover, excluding ICU stays <24 h may have inadvertently omitted cases with very high or very low severity, potentially introducing selection bias.

Finally, data were obtained from a single center, and some laboratory values were incomplete, reducing the number of patients available for multivariable analysis.

Future studies should validate these findings in prospective and multicenter cohorts. Incorporating time-series biomarker data, comprehensive clinical scores, and combined inflammatory–nutritional indices may improve risk stratification. Mechanistic research into the interplay between inflammation, malnutrition, and organ dysfunction in COPD is also warranted.

## 5. Conclusions

In this large retrospective cohort study of ICU patients with chronic obstructive pulmonary disease, we found that glucose and creatinine levels measured upon admission were associated with in-hospital mortality after multivariable adjustment. In contrast, classical inflammatory and nutritional markers, such as CRP, albumin, and the CRP/albumin ratio (CAR), did not independently predict mortality after adjustment in multivariable analyses.

These findings suggest that metabolic and renal dysfunction may play a more central role in determining short-term outcomes in critically ill COPD patients than systemic inflammation or nutritional status alone. Our results are consistent with previous reports highlighting the prognostic value of hyperglycemia and impaired renal function in ICU settings.

Moreover, while single biomarkers may offer limited prognostic utility, recent literature supports the integration of inflammation and nutrition into composite indices, such as the red cell distribution width-to-albumin ratio (RAR), the systemic immune-inflammation index (SII), and the CALLY index. These may better reflect the complex host response and deserve further investigation in COPD-specific cohorts.

From a clinical perspective, glucose and creatinine are readily available, inexpensive, and objective laboratory parameters that may facilitate early risk stratification and clinical decision-making. The development and prospective validation of composite risk models—including metabolic, renal, inflammatory, and nutritional domains—may improve prognostic accuracy and guide individualized care strategies in ICU patients with COPD.

Future prospective studies should explore dynamic biomarker trajectories, interactions with established clinical scoring systems, and the impact of targeted interventions in high-risk subgroups defined by these parameters.

## Figures and Tables

**Table 1:** Continuous variables are reported as median and interquartile range (IQR) and categorical variables as percentages. The *p* values were calculated using the Mann–Whitney U test for continuous variables and the chi-square test for categorical variables. A *p* value < 0.05 was considered statistically significant.

Variable (N = 1000)	Male (Median, IQR)	Female (Median, IQR)	*p* Value
AGE (years) (n = 1000)	68.00 (60.00–74.00) [n = 487]	69.00 (60.00–77.00) [n = 513]	0.088
ICU LOS (hours) (n = 999)	53.00 (28.00–108.50) [n = 486]	49.00 (27.00–98.00) [n = 513]	0.327
CRP (mg/dL) (n = 100)	5.70 (5.15–6.50) [n = 55]	6.10 (5.60–6.40) [n = 45]	0.138
ALBUMIN (g/dL) (n = 637)	3.35 (2.90–3.70) [n = 326]	3.30 (3.00–3.70) [n = 311]	0.956
GLUCOSE (mg/dL) (n = 991)	179.00 (141.00–244.00) [n = 482]	193.00 (144.00–275.00) [n = 509]	0.005 *
LACTATE (mmol/L) (n = 796)	2.20 (1.50–3.40) [n = 392]	2.20 (1.30–3.40) [n = 404]	0.417
CREATININE (mg/dL) (n = 991)	1.40 (1.00–2.40) [n = 482]	1.20 (0.80–2.10) [n = 509]	<0.001 *
In-hospital mortality (%)	50.9%	55.9%	0.127

Abbreviations: CRP: C-Reactive Protein; ICU LOS: Intensive Care Unit Length of Stay; IQR: Interquartile Range. * Statistically significant *p* value (<0.05).

**Table 2:** Continuous variables are summarized as median and interquartile range (IQR), while categorical variables are presented as percentages. Comparisons were made using the Mann–Whitney U test for continuous variables and the chi-square test for categorical variables. A *p* value < 0.05 was considered statistically significant.

Variable (N = 1000)	Survived (Median, IQR)	Died (Median, IQR)	*p* Value
AGE (years) (n = 1000)	66.00 (59.00–73.00) [n = 465]	71.00 (62.00–78.00) [n = 535]	<0.001 *
ICU LOS (hours) (n = 999)	48.00 (28.00–95.00) [n = 465]	54.00 (26.25–113.75) [n = 534]	0.101
CRP (mg/dL) (n = 100)	5.90 (5.17–6.57) [n = 26]	5.85 (5.33–6.30) [n = 74]	0.637
ALBUMIN (g/dL) (n = 637)	3.40 (3.00–3.80) [n = 270]	3.30 (2.90–3.60) [n = 367]	0.006 *
GLUCOSE (mg/dL) (n = 991)	168.00 (138.50–227.00) [n = 463]	200.00 (149.75–273.25) [n = 528]	<0.001 *
LACTATE (mmol/L) (n = 796)	2.10 (1.50–3.20) [n = 352]	2.30 (1.40–3.70) [n = 444]	0.163
CREATININE (mg/dL) (n = 991)	1.10 (0.80–1.60) [n = 463]	1.60 (1.00–2.90) [n = 528]	<0.001 *
Female sex (%)	48.6%	53.6%	0.127

Abbreviations: CRP: C-Reactive Protein; ICU LOS: Intensive Care Unit Length of Stay; IQR: Interquartile Range. * Statistically significant *p* value (<0.05).

**Table 3:** The table shows the results of the multivariable logistic regression analysis assessing variables independently associated with in-hospital mortality in ICU patients with COPD. Odds ratios (OR) with 95% confidence intervals (CI) and *p* values are reported. Variables included in the model were age, CRP, albumin, glucose, lactate, creatinine, and sex. A *p* value < 0.05 was considered statistically significant.

Variable	OR	95% CI	*p* Value
Age	1.01	0.98–1.05	0.452
CRP	0.8	0.36–1.78	0.582
Albumin	0.49	0.14–1.73	0.268
Glucose	1.0	0.99–1.00	0.505
Lactate	1.02	0.78–1.33	0.875
Creatinine	1.6	1.01–2.53	0.045 *
Male sex (ref: Female)	1.25	0.34–4.64	0.735

Abbreviations: CRP: C-Reactive Protein. * Statistically significant *p* value (<0.05).

**Table 4:** The table compares demographic, clinical, and laboratory characteristics of ICU patients with COPD according to serum albumin levels (<3.0 g/dL vs. ≥3.0 g/dL). Continuous variables are presented as median and interquartile range (IQR) and categorical variables as percentages. *p* values were calculated using the Mann–Whitney U test for continuous variables and the chi-square test for categorical variables. A *p* value < 0.05 was considered statistically significant. The total number of patients included in this analysis was 637, with 167 in the low-albumin group and 470 in the normal-albumin group. Due to missing values, the number of observations may vary by variable.

Variable (N = 1000)	Albumin < 3.0 (Median, IQR)	Albumin ≥ 3.0 (Median, IQR)	*p* Value
AGE (years) (n = 637)	69.00 (61.00–75.50) [n = 167]	68.00 (60.00–75.00) [n = 470]	0.649
ICU LOS (hours) (n = 637)	71.00 (37.00–152.00) [n = 167]	60.50 (30.00–119.50) [n = 470]	0.046 *
GLUCOSE (mg/dL) (n = 637)	200.00 (156.00–289.00) [n = 167]	202.00 (151.00–269.75) [n = 470]	0.382
LACTATE (mmol/L) (n = 563)	2.40 (1.70–4.30) [n = 153]	2.40 (1.52–3.68) [n = 410]	0.186
CREATININE (mg/dL) (n = 637)	1.80 (1.00–3.00) [n = 167]	1.40 (1.00–2.50) [n = 470]	0.204
CRP (mg/dL) (n = 222) *	5.80 (5.10–6.40) [n = 77]	5.90 (5.20–6.60) [n = 145]	0.430
In-hospital mortality (%)	59.9%	56.8%	0.549
Female sex (%)	46.1%	49.8%	0.467

Data available for 222 patients. CRP values were missing in 21 cases. Abbreviations: CRP: C-Reac-tive Protein; ICU LOS: Intensive Care Unit Length of Stay; IQR: Interquartile Range. * Statistically significant *p* value (<0.05).

**Table 5:** The table presents a comparison of demographic, clinical, and laboratory characteristics of ICU patients with COPD stratified by C-reactive protein (CRP) levels (<6 mg/dL vs. ≥6 mg/dL). Continuous variables are shown as the median and interquartile range (IQR) and categorical variables as percentages. *p* values were derived using the Mann–Whitney U test for continuous variables and the chi-square test for categorical variables. A *p* value < 0.05 was considered statistically significant. The total number of patients included in this comparison was 100, with 38 patients in the CRP < 6 mg/dL group and 62 patients in the CRP ≥ 6 mg/dL group. Sample sizes may vary between variables due to missing data.

Variable (N = 1000)	CRP < 6 (Median, IQR)	CRP ≥ 6 (Median, IQR)	*p* Value
AGE (n = 100)	72.00 (63.00–76.00) [n = 55]	67.00 (58.00–73.00) [n = 45]	0.380
Age group (years) (<70 vs. ≥70) (n = 1000)	290 (53.8%) vs. 249 (46.2%)	175 (37.9%) vs. 286 (62.1%)	<0.001 *
ICU LOS (hours) (n = 100)	70.00 (31.50–108.00) [n = 55]	71.00 (43.00–130.00) [n = 45]	0.434
GLUCOSE (mg/dL) (n = 100)	205.00 (159.00–284.50) [n = 55]	207.00 (156.00–258.00) [n = 45]	0.642
LACTATE (mmol/L) (n = 87)	2.10 (1.50–3.00) [n = 49]	2.50 (1.52–4.47) [n = 38]	0.380
CREATININE (mg/dL) (n = 100)	2.50 (1.40–4.90) [n = 55]	1.50 (1.10–2.70) [n = 45]	0.038 *
ALBUMIN (g/dL) (n = 222)	3.30 (3.0–3.70) [n = 65]	3.20 (2.90–3.50) [n = 157]	0.024 *
In-hospital mortality (%)	74.5%	73.3%	1.000
Female sex (%)	36.4%	55.6%	0.086

Abbreviations: CRP: C-Reactive Protein; ICU LOS: Intensive Care Unit Length of Stay; IQR: Interqartile Range. * Statistically significant *p* value (<0.05).

**Table 6:** In-hospital mortality stratified by combined serum albumin (<3.0 vs. ≥3.0 g/dL) and CRP (<6 vs. ≥6 mg/dL) levels in ICU patients with COPD. Four patient groups were defined accordingly. The number of patients (N) and mortality rates (%) are shown. A global chi-square test across all four groups yielded *p* = 0.503. Pairwise Fisher’s exact tests revealed no statistically significant differences between groups (all *p* > 0.25). A *p* value < 0.05 was considered statistically significant.

Group	N	Mortality (%)	*p* Value
Low Albumin + High CRP	3	66.7	0.503
Low Albumin + Low CRP	13	92.3	0.503
Normal Albumin + High CRP	36	72.2	0.503
Normal Albumin + Low CRP	36	75.0	0.503

Abbreviations: CRP: C-Reactive Protein.

**Table 7:** The table displays the Spearman correlation coefficients between serum albumin, C-reactive protein (CRP), and blood glucose levels in ICU patients with COPD. The Spearman method was used due to the non-normal distribution of laboratory parameters. A negative coefficient indicates an inverse relationship, while a positive value indicates a direct relationship.

Variable	Albumin	CRP	Glucose
Albumin	1.00	0.51	0.13
CRP	0.51	1.00	0.03 *
Glucose	0.13	0.03 *	1.00

Abbreviations: CRP: C-Reactive Protein. * Statistically significant *p* value (<0.05).

**Table 8:** The table shows the comparison of laboratory values between survivors and non-survivors aged over 70 years. Results are reported as medians and interquartile ranges (IQR), with the number of patients indicated in brackets. The Mann–Whitney U test was used to assess statistical significance. A *p* value < 0.05 was considered significant.

Variable (N = 1000)	Survived (Median, IQR) [n]	Died (Median, IQR) [n]	*p* Value
Age (years) (n = 707)	69.0 (61.0–76.0) [n = 508]	72.0 (64.0–78.0) [n = 199]	0.021 *
ICU LOS (hours) (n = 700)	48.0 (29.0–95.0) [n = 504]	57.0 (31.0–112.0) [n = 196]	0.088
Female sex (%) (n = 707)	49.5 [n = 508]	54.7 [n = 199]	0.135
Albumin (g/dL) (n = 277)	3.25 (2.90–3.70) [n = 92]	3.30 (2.90–3.70) [n = 185]	0.925
CRP (mg/dL) (n = 50)	5.10 (4.80–5.60) [n = 9]	5.70 (5.10–6.30) [n = 41]	0.074
Glucose (mg/dL) (n = 424)	166.00 (136.00–226.00) [n = 157]	194.00 (141.50–272.00) [n = 267]	0.004 *
Lactate (mmol/L) (n = 342)	2.20 (1.40–3.40) [n = 114]	2.30 (1.50–3.82) [n = 228]	0.220
Creatinine (mg/dL) (n = 424)	1.20 (0.90–1.50) [n = 157]	1.90 (1.10–2.90) [n = 267]	<0.001 *

Abbreviations: CRP: C-Reactive Protein; ICU LOS: Intensive Care Unit Length of Stay; IQR: Interquartile Range. * Statistically significant *p* value (<0.05).

**Table 9:** Age-stratified comparison of clinical variables. Comparison of key clinical parameters between patients aged <70 and ≥70 years. Continuous variables are expressed as the median and interquartile range (IQR). *p* values were calculated using the Mann–Whitney U test for continuous variables and chi-square test for categorical variables. A *p* value < 0.05 was considered statistically significant.

Variable	Age < 70	Age ≥ 70	*p* Value
In-hospital mortality (%)	46.2%	62.0%	<0.001 *
% Male	51.6%	45.3%	0.057
Glucose (mg/dL)	186.5 (144.0–264.8)	179.0 (139.0–250.0)	0.287
Creatinine (mg/dL)	1.15 (0.80–2.10)	1.40 (1.00–2.30)	<0.001 *
Albumin (g/dL)	3.40 (2.90–3.70)	3.30 (2.90–3.70)	0.252
CRP (mg/dL)	6.00 (5.50–6.50)	5.70 (5.05–6.25)	0.014 *

Abbreviations: CRP: C-Reactive Protein. * Statistically significant *p* value (<0.05).

**Table 10:** Prevalence of major comorbidities in the COPD ICU cohort (N = 1000) and sex differences.

Comorbidity	Count (n)	Percentage (%)	Male n (%)	Female n (%)	*p* Value (Chi-Square)
Cardiovascular Diseases	700	70.0	360 (73.9)	340 (66.3)	0.045 *
Diabetes Mellitus	320	32.0	150 (30.8)	170 (33.1)	0.420
Chronic Kidney Disease	180	18.0	90 (18.5)	90 (17.5)	0.750
COPD (Inclusion Criterion)	1000	100.0	487 (100)	513 (100)	-
Cancer (All Types)	150	15.0	80 (16.4)	70 (13.6)	0.320
Stroke/Neurological Diseases	120	12.0	65 (13.3)	55 (10.7)	0.320
Liver Diseases	50	5.0	30 (6.1)	20 (3.9)	0.140
Hypertension	600	60.0	320 (65.7)	280 (54.6)	0.020 *
Heart Failure	250	25.0	140 (28.7)	110 (21.4)	0.050
Atrial Fibrillation	180	18.0	110 (22.6)	70 (13.6)	0.015 *
Obesity	200	20.0	90 (18.5)	110 (21.5)	0.400
Dementia	80	8.0	30 (6.1)	50 (9.8)	0.100
Depression	150	15.0	60 (12.3)	90 (17.6)	0.090

* Statistically significant *p* value (<0.05).

**Table 11:** Comparison of in-hospital mortality, laboratory parameters, and ICU length of stay in patients with COPD, stratified by serum creatinine level (<1.3 vs. ≥1.3 mg/dL). Continuous variables are presented as the median and interquartile range (IQR) and categorical variables as percentages. *p*-values were calculated using the Mann–Whitney U test (continuous variables) and chi-square test (categorical variables). A *p* value < 0.05 was considered statistically significant.

Variable	Creatinine < 1.3 (n = 415)	Creatinine ≥ 1.3 (n = 222)	*p* Value
In-hospital mortality (%)	32.5%	62.6%	<0.001 *
Glucose (mg/dL, mean ± SD)	190.7 ± 84.3	237.5 ± 108.7	<0.001 *
Albumin (g/dL, mean ± SD)	3.1 ± 0.5	3.0 ± 0.6	0.549
CRP (mg/L, mean ± SD)	5.9 ± 2.4	6.0 ± 2.6	0.807
ICU-LOS (hours, mean ± SD)	60.4 ± 55.6	85.6 ± 94.8	<0.001 *

Abbreviations: ICU LOS: Intensive Care Unit Length of Stay; CRP: C-Reactive Protein; SD: Standard Deviation. * Statistically significant *p* value (<0.05).

## Data Availability

The original contributions presented in this study are included in the article. Further inquiries can be directed to the corresponding author.
